# Hepatic Arterial Buffer Response in Liver Radioembolization and Potential Use for Improved Cancer Therapy

**DOI:** 10.3390/cancers13071537

**Published:** 2021-03-26

**Authors:** Stephan Walrand, Michel Hesse, Philippe d’Abadie, François Jamar

**Affiliations:** Nuclear Medicine Department, Cliniques Universitaires Saint-Luc, 1200 Brussels, Belgium; michel.hesse@uclouvain.be (M.H.); philippe.dabadie@uclouvain.be (P.d.); francois.jamar@uclouvain.be (F.J.)

**Keywords:** liver radioembolization, cancer therapy, dose optimization, TARE, SIRT

## Abstract

**Simple Summary:**

Radioembolization of hepatic tumors is performed by injecting ^90^Y or ^166^Ho loaded spheres into the hepatic artery. A twofold tumor to normal liver absorbed dose ratio is commonly obtained. In order to improve tumoral cell killing while preserving lobule function, co-injection of arterial vasoconstrictor has been proposed, but without success: the hepatic arterial buffer response quickly inhibits the arterioles vasoconstriction. The aim of the study is to investigate whether it is possible to take benefit from this buffer response, by co-infusing a mesenteric arterial vasodilator in order to dump the hepatic lobules arterial flow. Animal studies evidencing such mechanism are reviewed. Some potential mesenteric vasodilators are identified and their safety profile discussed. A four to sixfold improvement of the tumoral to normal tissue dose ratio is expected, pushing the therapy towards a real curative intention, especially in hepatocellular carcinoma (HCC), more frequent in obese subjects, and where ultra-selective spheres delivery is often not possible.

**Abstract:**

Liver radioembolization is a treatment option for unresectable liver cancers, performed by infusion of ^90^Y or ^166^Ho loaded spheres in the hepatic artery. As tumoral cells are mainly perfused via the liver artery unlike hepatic lobules, a twofold tumor to normal liver dose ratio is commonly obtained. To improve tumoral cell killing while preserving lobules, co-infusion of arterial vasoconstrictor has been proposed but with limited success: the hepatic arterial buffer response (HABR) and hepatic vascular escape mechanism hamper the arterioles vasoconstriction. The proposed project aims to take benefit from the HABR by co-infusing a mesenteric arterial vasodilator: the portal flow enhancement inducing the vasoconstriction of the intra sinusoids arterioles barely impacts liver tumors that are mainly fed by novel and anarchic external arterioles. Animal studies were reviewed and dopexamine was identified as a promising safe candidate, reducing by four the hepatic lobules arterial flow. A clinical trial design is proposed. A four to sixfold improvement of the tumoral to normal tissue dose ratio is expected, pushing the therapy towards a real curative intention, especially in HCC where ultra-selective spheres delivery is often not possible.

## 1. Introduction

Liver radioembolization using ^90^Y or ^166^Ho loaded spheres injected via the hepatic artery is an evolving technique for primary and secondary liver tumors treatment [1]. Normal liver tissue is mainly fed by the portal vein. On the contrary, tumors, in order to sustain their high metabolism, trigger arterial angiogenesis by releasing vascular endothelial growth factor (VEGF). As a result, a tumor to normal tissue dose ratio (T/N) of about 2.1 ± 1.3 and 1.8 ± 0.9 are commonly reached in hepatocellular carcinoma (HCC) and in secondary colorectal cancer (CRC) tumors [2], respectively.

The maximal tumor dose reachable is thus limited by the need to preserve the remaining functional liver tissue. Contrary to external beam radiotherapy (EBRT), where uniform dose deposition is feasible, the tumor-absorbed doses in radioembolization are very heterogeneous due to the random nature of sphere dynamic transport in the arterial tree [3]. As a result, tumor-absorbed doses above 200 Gy are required in order to get significant patient outcome improvement [4].

Similarly to other radionuclide therapies, after their release, the spheres cannot be guided preferably to the tumors with certainty. Patients with a few solitary tumors can be treated at a segmental level, permitting to achieve an ultra-selective sphere delivery by successively setting the catheter tip in the segment regions containing the tumors. For hepatocellular carcinoma (HCC), liver radioembolization is often performed for advanced stages with large and multifocal lesions and the spheres must be delivered in a large part of the liver [5].

In order to increase T/N, continuous co-infusion of arterial vasoconstrictor via the catheter line has been proposed but with limited effect [6,7,8]. Indeed, when the arterial flow is too low, the induced shear stress releases nitric oxide, which re-dilates the arterioles, a mechanism named vascular escape [9]: the arterial vasoconstriction is hampered and the nominal arterial flow is recovered within a few minutes despite the continuing vasoconstrictor infusion.

Beside the vascular escape mechanism, the hepatic lobules are also able to reduce their arterial flow when the portal flow increases in order to keep the total flow constant, a mechanism named hepatic arterial buffer response (HABR) [10].

The goal of the present study is to investigate how benefit could be taken from the HABR by co-infusing a mesenteric arterial vasodilator. Due to the resulting portal flow increase, the functioning hepatic lobules will reduce their arteriole flow, reducing by the way the number of spheres trapped in their triads as previously observed during the first minute of arterial vasoconstrictor co-infusion [6,7,8]. Literature was reviewed in order to identify such a safe mesenteric arterial vasodilator and its impact on triads spheres trapping. Recent knowledges on HABR and HCC tumors angiogenesis are discussed in order to predict the improvement on T/N. Intra-subject assessment of this improvement by dual isotope single photon emission tomography/computed scan (SPECT/CT) is proposed. Dopexamine was identified to be safe and to promise a sixfold T/N improvement which should push the therapy to a real curative intent.

## 2. T/N Improvement Trials Using Arterial Vasoconstrictor

van den Hoven et al. [11] performed a systematic review of T/N improvement trials using a vasoconstrictor. Six trials [12,13,14,15,16,17] investigating the utilization of angiotensin-II (AT-II) in liver radioembolization with ^90^Y loaded spheres were found. These studies used a hepatic arterial bolus of 20 or 50 μg AT-II about 30 s before the radioembolization. None of these studies exhibited any efficacy.

Three other studies [6,7,8] using continuous 10 μg/min AT-II hepatic arterial infusion reported potential T/N median improvement factor ranging from 1.8 to 3.1. However, none of these studies was performed in realistic clinical therapy conditions, and no other studies were found since this review.

Goldberg et al. [6,7] compared ^99m^Tc-MAA and ^131^I-MAA radioembolization in colorectal metastases performed before and just after a 100 s AT-II infusion. The MAA were injected in 10 s, which is challenging to perform in therapy sessions, and impossible when several tip positions are used.

**Figure 1:**
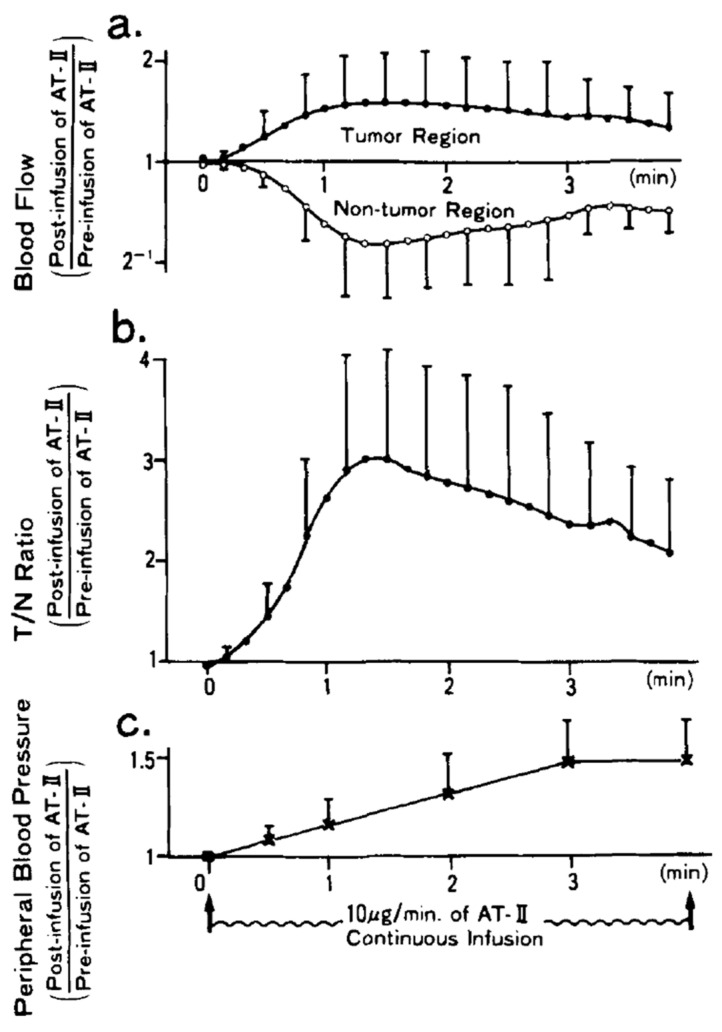
(**a**) Arterial blood flow, (**b**) tumor to normal tissue dose ratio (T/N) improvement, and (**c**) peripheral blood pressure during a continuous 10 μg/min AT-II arterial infusion (reprinted from [8] with authorization of Willey and Sons). Note the decrease of T/N improvement after 80 s despite the continuing AT-II infusion.

The study of Sasaki et al. [8] is very interesting regarding the impact of the radioembolization duration. This study monitored the tumoral and non-tumoral arterial blood flow using a gamma camera, by continuously infusing a 5% glucose solution loaded with the short half-life ^81m^Kr isotope. Figure 1 shows the different parameters monitoring. Figure 1 shows that a T/N maximal improvement of three is obtained after about 80 s explaining the T/N improvement observed by Goldberg et al. [6,7]. However, despite the continuing AT-II infusion, the arterial flow restarts to increase: a mechanism named hepatic vascular escape (see Section 4.1.2). As a result, the T/N improvement decreases and seems to vanish about 7 min after starting the infusion.

## 3. Hepatic Arterial Flow Reduction from HABR Triggering

### 3.1. Physiological Postprandial HABR

Unlike the AT-II vasoconstriction, hepatic lobule arterioles constriction is sustained by the HABR as long as the portal flow is increased.

**Figure 2:**
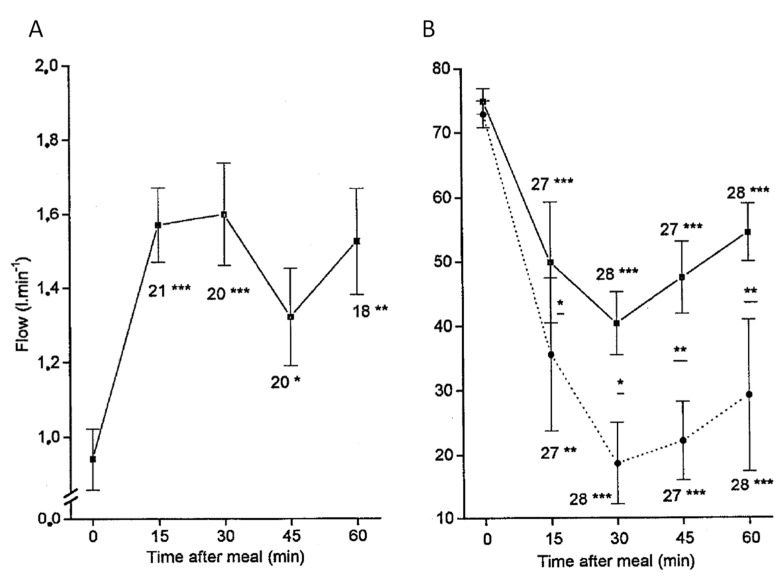
(**A**) Portal flow and (**B**) arterial to portal blood velocity ratio, both monitored by Doppler ultrasonography after ingestion of a standard liquid meal of 500 kcal. In (**B**), full and dashed curves correspond to left and right hepatic arteries, respectively. The number of observations and *p* for the student *t*-test for each measurement against the baseline appear under the error bars: * *p* < 0.05; ** *p* < 0.005; *** *p* < 0.0005. (reprinted from [18] with authorization of Springer Nature).

Figure 2 shows the power of the HABR mechanism in a healthy subject during meal digestion: while the portal flow is increased by a factor 1.6 to bring the nutrients, the left and right hepatic arterial flows are reduced by a factor 1.15 and 2.5, respectively. The authors note that this discrepancy could be explained by differences in diameter changes that the doppler techniques are not able to measure. Note that there are a few minutes delay for the arterial flow stabilization, which can be seen from the continuous adaptation between 15 to 30 min although the portal flow is constant. More importantly, the arterial flow reduction is sustained as long as the portal flow is increased. Taking into account that the tumor flow will increase by a factor 1.5 due to the arterial flow redirection (Figure 2A), the potential T/N could reach a factor 3 in the right liver.

### 3.2. HABR Triggering with Sodium Acetate Infusion

**Figure 3:**
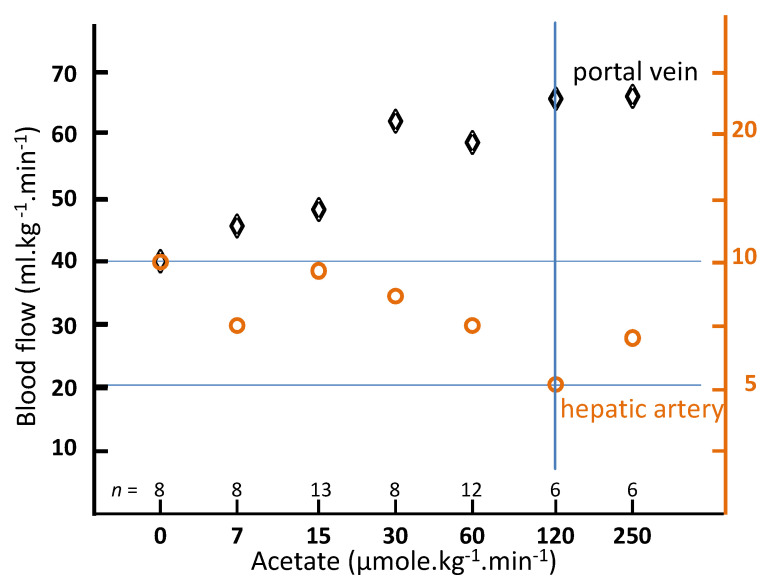
Portal and arterial hepatic blood flow in conscious rats as a function of the acetate infusion rate after 10 min of infusion (derived from [19]).

Carmichael et al. [19] studied the impact of intra-venous sodium acetate infusion on the hepatic blood flow in rats. A cannula was inserted into the left ventricle under anesthesia. Two hours after waking up, radioactive 15 μm-diameter spheres were injected in the left ventricle over a period of 20 s by use of an infusion pump. The spheres injection was performed in control rats (*n* = 18) and also 10 min after starting sodium acetate infusion, ranging from 7 to 250 μmol·kg^−1^·min^−1^ through the jugular vein. During the whole experiment, the rectal temperature was maintained at 37 °C with heating lamps. After spheres infusion, the rats were sacrificed and the organs counted, the liver counts representing the hepatic arterial flow, while the portal flow was given by the sum of spleen, stomach, and bowel counts. Figure 3 shows the hepatic portal and arterial flow as a function of the sodium acetate infusion rate. A twofold arterial flow reduction was observed for a 120 μmol·kg^−1^·min^−1^ infusion rate.

In the mesenteric system, only the intestines exhibited noticeable blood flow increase, i.e., +90% for the small intestine and +50% for the large intestine, both at 120 μmol·kg^−1^·min^−1^. A small increase of about 18% of the coronary spheres trapping was observed. Based on these observations and by repeating experiments with co-infusing different adenosine agonists and blockers, the author concluded that the acetate metabolism by intestine tissue locally produces adenosine in the interstitial space resulting in a vasodilatation of the pre-portal system.

The portal flow stagnation, joined with an artery flow increase from 120 to 250 μmol·kg^−1^·min^−1^, is compatible with about 5% of spheres passing throughout the intestine capillaries due to their high dilatation: 3% of the spheres having a diameter close to 9 μm. Small increases of the hepatic arterial flow observed in other studies at high acetate rate in rats [20] or in dogs using the same sphere diameter range [21] could result from this intestine sphere leakage. The key point is that these two studies evidenced a large portal flow increase.

Beside rat studies that are sufficient for accurate liver flow assessment, preclinical dose-toxicity studies are often performed in dogs having a global metabolism closer to that of humans. One sodium acetate infusion study in dogs [22] reported no adverse side effect with 1000 μmol·kg^−1^·min^−1^ infusion rate during 10 min in 5 dogs.

Table 1 shows acetate infusion rate and duration performed in human volunteers for metabolism studies. No adverse side effect was reported.

The acetate infusion studies in healthy volunteers proved the dose rate required for T/N improvement to be safe. A 2 mmole/mL sodium acetate for intravenous injection after dilution is registered at the FDA [28]. Contraindications are hypernatremia or fluid retention. The solution has to be used with great care in patients with congestive heart failure, renal insufficiency, metabolic, or respiratory alkalosis, and severe hepatic insufficiency. Acetate was used for a long time as a buffer for metabolic acidosis and in haemodialysis baths [29].

Potential side effects of sodium load are overhydration, congested states, or pulmonary edema. Acetate could induce an asymptomatic and transient metabolic alkalosis during an acute intravenous infusion. No other clinicals effects were demonstrated in normal subjects and especially, no changes in cardiac pulses and blood pressure [30].

### 3.3. Arterial Flow Reduction Using Dopexamine

Several vasoactive agents have been shown to restore the portal flow in septic shock, traumatic shock, and cirrhosis [31,32,33,34,35].

Amongst them, dopexamine, a synthetic analogue of dopamine, has the advantage to induce vasodilation through the β_2_ adrenoceptors and more specifically through the dopamine DA_1_ receptors especially present in the renal, coronary, and mesenteric arteries [31].

Dopexamine infusion was showed to increase by an impressive factor the portal flow in twelve healthy volunteers during 3 μg/kg/min infusion, but without significant change in the hepatic arterial flow [35]. However, the low postprandial hepatic arterial flow reduction observed in this study makes questionable accuracy of the arterial flow monitoring performed by Doppler sonography.

In a more robust hepatic arterial flow assessment using radioactive microspheres as described in previous section, an impressive fourfold hepatic arterial flow reduction was observed during 3.6 μg/kg/min dopexamine infusion in six-dog cohorts [36], while in a similar setup, only a twofold reduction was observed using dopamine [37]. Table 2 shows that the pre-portal organ exhibiting the highest increase was the stomach. Like in acetate studies, the re-increase of the liver uptake at high dose rate (last table column) likely results from a spheres leakage due to the high pre-portal capillaries vasodilation.

Intravenous dopexamine infusion is used in hospitals to treat heart failure, especially following cardiac surgery [38]. Dopexamine is characterized by a rapid onset and a short duration of action: on termination of infusion the plasma concentration decreases with a half-life of 7 min. Dopexamine is well tolerated in infusion rate below 10 μg/kg/min and during less than 72 h. The most disturbing adverse effects during a catheterization should be vomiting (3.7%) and tremors (2.5%). Other adverse effects are nausea (5.3%), chest pain/angina pectoris (2.1%), ventricular extrasystoles (2.3%), hypotension (2.1%), atrial fibrillation (1.4%), and hypertension (1.4%). All effects rapidly responded to rate reduction or infusion termination. Dopexamine is contraindicated in patients with thrombocytopenia, and caution is advised in case of hyperglycemia and of hypokalemia.

Since 2010, dopexamine in cardiac emergencies was progressively replaced by other drugs more appropriate for this purpose, such as epinephrine, dopamine, dobutamine, norepinephrine, and levosimendan.

## 4. Potential Effect on T/N

### 4.1. Liver Vascular Flow Regulation Mechanisms

The liver plays a major role in the clearance of many drugs and hormones, which are hepatic blood-flow-dependent. To allow a fine control of blood levels by the endocrine glands, the liver has multiples mechanisms acting on different time scales to maintain the hepatic blood flow constant [10]; we will focus on the two of interest in radioembolization.

#### 4.1.1. HABR

The HABR is the principal flow regulation mechanism and is in charge to manage the portal flow increases which occur during hourly periods several times per day, as a result of the nutrients digestion.

The human liver is made of about 10^6^ lobules independently working in parallel: each lobule can be considered as a full miniature liver regulating by itself its behavior in function of its entering blood flow. The lobule is a hexagonal prism of 1 mm diameter by 1.5 mm length: at each corner of any lobule is located a triad containing an artery, a portal vein, and a bile duct. Arterial and portal blood are mixed in the space of Mall just before entering the sinusoids in which the hepatic cells extract and metabolize the nutrients, some metabolites being eliminated via the bile duct. Afterwards the cleared blood is collected in the central vein.

**Figure 4:**
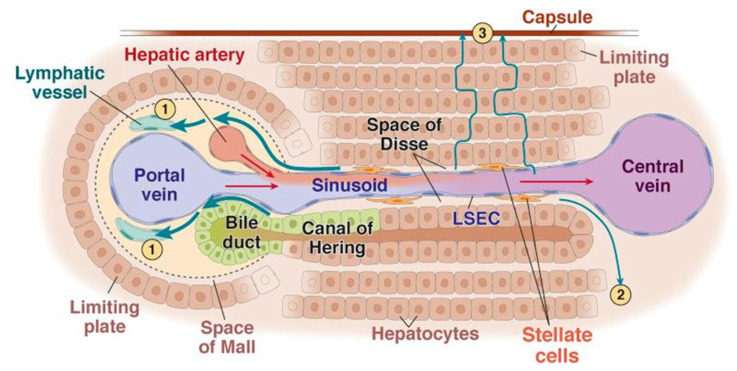
Schematic representation of the intra lobule vascularization to the central vein from one of the six triads. 1,2,3: lymphatic fluid flows. (reprinted from [39] with permission of Elsevier).

In the space of Mall (Figure 4), specialized cells continuously produce adenosine in an oxygen-independent way. A decrease in portal flow results in reduced adenosine washout, leading to a vasodilatation of the triad artery which constitutes the HABR mechanism [10].

#### 4.1.2. Hepatic Vascular Escape

When the arterial flow is reduced by nervous system action or by a vasoconstrictor, the adenosine washout in the space of Mall is also reduced and the increasing adenosine concentration tends to re-dilate the triad artery, hampering the way of the vasoconstrictor action. After a while, the shear stress induced by the vasoconstriction induces a nitric oxide release which inhibits the vasoconstrictor action and results in a return to the nominal arterial blood flow [9,10]. The combined effect of these two mechanisms is clearly illustrated in Figure 1: a maximal arterial flow reduction lower than that achieved using dopexamine infusion and a return to the nominal flow in a few minutes despite the continuous AT-II infusion.

### 4.2. HABR in Cirrhotic Liver

Cirrhosis is the major risk factor for HCC and represent about 80% of patients with newly diagnosed HCC. This points out the importance of analyzing the HABR in cirrhotic liver.

The key point to keep in mind for the current purpose of the HABR is that it is an intra-lobule mechanism, and is thus likely available in any healthy hepatic lobule, even if the whole cirrhotic liver could present an altered global HABR.

**Figure 5:**
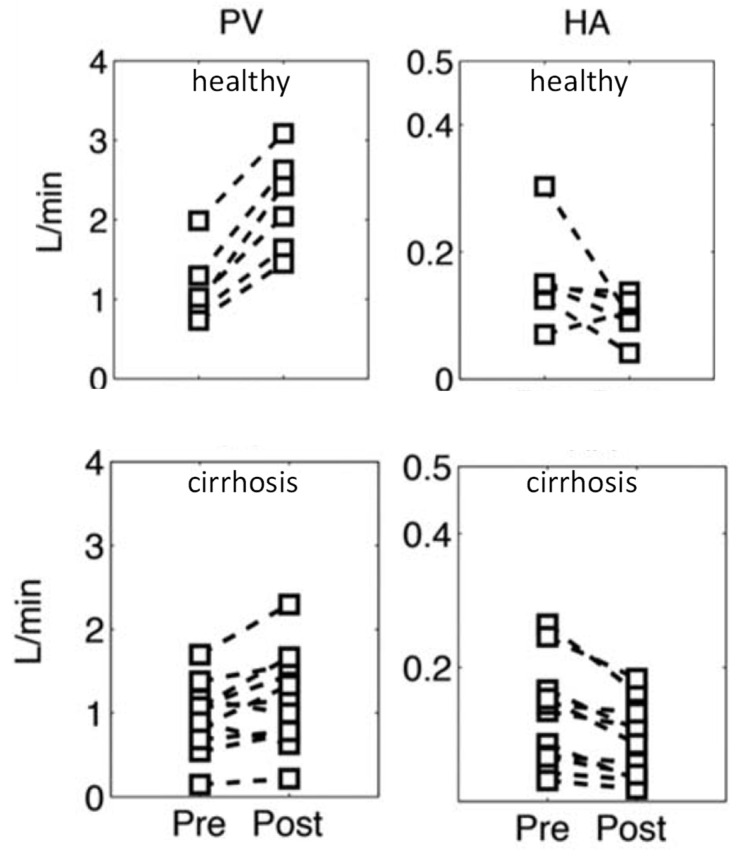
Changes in portal flow (PV) and in hepatic artery flow (HA) post-ingestion of a 700 kcal meal in six healthy volunteers and 12 cirrhotic patients measured by 4D flow MRI (reprinted from [42] with permission of Willey and Sons). Note that the data correspond to the global arterial flow; healthy lobules likely underwent a higher arterial flow reduction.

Amazingly, global HABR, which is the sum of all individual lobules HABR (parallel circuit), is often maintained in cirrhotic livers with or without portal hypertension [40,41]. Roldan-Alzate et al. [42] performed a 4D flow MRI study on six volunteers and 12 patients with portal hypertension linked to cirrhosis. Figure 5 shows the portal flow (PV) and hepatic artery flow (HA) before and 20 min after a 700 kcal meal ingestion. Although the postprandial portal flow increase is lower in the cirrhotic group, these patients still exhibit a postprandial global hepatic arterial flow reduction. This supports that the HABR is preserved in the remaining healthy hepatic lobules.

No study investigating the impact of dopexamine on portal flow in cirrhosis was found. However, other less mesenteric-specific vasodilators have been shown to increase the portal flow in cirrhosis model [43] and in cirrhotic patients as well [34,44,45,46,47]. 

### 4.3. Hepatic Tumor Vascularization

Secondary liver tumors do not derive from the hepatic lobule, but from circulating primary cancer cells being trapped in the hepatic sinusoids, in which many are killed by the natural killer (NK) and Kuppfer’s cells [48]. Some of them can escape by crossing the sinusoidal fenestration to form a micro-metastasis in between the hepatocytes. Promptly, these micro-metastases release VEGF to promote arterial angiogenesis in order to sustain their high metabolism. The resulting anarchic and immature arterial vascularization does not have any smooth muscle able to produce vasoconstriction as proved by the three AT-II studies [6,7,8], and thus will not respond to portal flow increase. 

This is also the case for advanced HCCs which constitute a significant fraction of radioembolizations and in which tumors are abundantly supplied by neo-arteries independent of triad flow as a result of sinusoidal capillarization [49,50,51].

The situation is more complex in early HCC stage where tumors of diameter less than 3 cm often receive preferential portal venous blood [51]. No study investigating whether the arterial vasculature of these tumors respond to a portal flow increase was found.

## 5. Animal Models for T/N Improvement Assessment

The continuously increasing reinforcement of ethical considerations could make it difficult to directly perform studies on humans, such as reported in Section 2 which already date from one or two decades. With this in regard, we also reviewed the animal liver tumors model reported in the literature. Unfortunately, no such usable animal model for T/N improvement evaluation was found. In Section 5.1, we give a lead to obtain such a model for metastatic T/N improvement evaluation, and in Section 5.2, we discuss the limitation of existing animal HCC model for the current purpose.

### 5.1. Metastatic Model

Ectopic spleen tissue masses are present in 16% of patients undergoing contrast-enhanced abdominal CT [52]. These tissues appear well-marginated and exhibit a clear re-enhancement during the arterial-phase mimicking tumors [53]. They belong to two different groups [54]. 

Accessory spleens result from lobule fusion failure during the fetal development that releases splenic cells in the circulation. After being trapped in capillaries, they continue the normal development during the fetal phase and end up in functional tissue, histologically similar to that of the normal spleen. 

**Figure 6:**
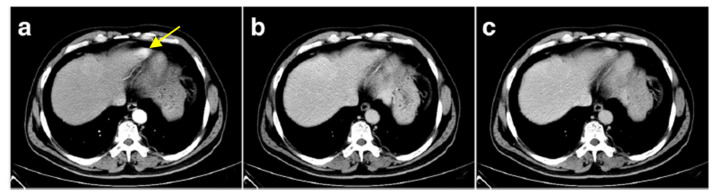
Contrast enhanced liver CT. (**a**–**c**): Arterial, portal, and equilibrium phase, respectively (reprinted from [55] under the license CC BY 4.0). Yellow arrow: left liver apex mass which re-enhanced contrast during the arterial phase.

Splenosis arises from traumatic splenic rupture or splenectomy. Splenosis is missing some splenic characteristics such as smooth muscle elements. Due to the trapping of the released spleen cells by the pre-portal system capillaries, intrahepatic splenosis is rare. Figure 6 shows a contrast-enhanced CT in a patient who underwent a splenectomy five years before following a high-altitude accident. The CT showed an intrahepatic mass in the left apex of the liver, evidenced as splenic cell tissue after surgical resection [55].

An animal model could be obtained by re-injecting, via the hepatic artery, splenic cells obtained by biopsy of its own spleen. Being an autograft, these cells will easily grow to produce intrahepatic splenosis mimicking liver metastasis. Indeed, being introduced via the hepatic artery, they will be independent of the portal flow, and thus independent of the HABR mechanism as well, and similarly to tumor, will be insensitive to vasoactive agents. Such animals would be helpful in drugs screening to determine the optimal one in metastatic tumor radioembolization.

### 5.2. HCC Model

These last decades, several HCC models in animals have been developed in order to improve our knowledge on HCC. Chemical induction, genetic engineering, xenograft mouse model, and patient-derived xenografts are used [56]. Induced HCC in rats by implementing rat hepatoma cell lines N1S1 [57,58,59], McA-RH7777 [60], CBRH-7919 [61] has recently been used to investigate trans-arterial embolization.

These models are valuable to investigate trans-arterial chemoembolization (TACE) or trans-arterial radioembolization (TARE) efficacy, especially in synergy with other drugs [58,59,60,61]. However, due to their very different development process versus that of obesity-related HCC in human, there is no assurance that their response to portal flow increase would be similar to that of HCC in humans.

## 6. Clinical Trial for T/N Improvement Assessment

Although postprandial portal flow enhancement associated with an arterial flow decrease was observed in HCC patients with portal hypertension [12], no similar study was found in HCC patient presenting a portal vein thrombosis. Part of the clinical study should be to perform a postprandial portal vein flow assessment by echo doppler or MRI to evaluate whether their enrolment in the trial is appropriate. 

The dopexamine efficiency to improve T/N in HCC can be assessed in patients during the MAA radioembolization simulation performed before the therapeutic session using a dual isotope SPECT/CT [6,7]. 

After the catheter tip being rightly positioned, the ^99m^Tc-MAA will be infused in the usual way, immediately followed by the continuous dopexamine infusion. When the continuous Doppler monitoring will show the end of the hepatic arterial blood velocity drop, ^131^I-MAA or ^111^In-MAA will be infused and the dopexamine infusion stopped. 

Rightly positioning the catheter tip to target the tumors is rather challenging and a recent study has discussed the different factors explaining imaging discordance sometimes observed between angiography and MAA-SPECT or spheres-PET [62]. For the present purpose, the key point will be to accurately keep the catheter tip at the same position between the two MAA injections. Importantly, the chosen position should be clearly away from artery bifurcation. 

Although never reported, triple isotope SPECT/CT using ^131^I-MAA, ^111^In-MAA, and ^99m^Tc-colloidal sulfur which is taken up only by healthy hepatic tissue [63], could be considered in order to get an accurate HCC tumors registration together with the regional MAA uptake change. The two MAA injections will have to be performed into the catheterization room without moving the catheter tip and will be imaged afterwards. On the other hand, if phantom studies reveal that the ^99m^Tc imaging is too impacted by the scattered ^131^I and ^111^In gamma rays, the 99mTc-colloidal injection and imaging could be performed before the MAA catheterization.

Maximal arterial flow reduction is obtained about 30 min post meal ingestion (Figure 2), while a higher reduction was already observed 10 min post acetate infusion starting (Figure 3). Unfortunately, the reduction as a function of time was not assessed for acetate. However, regarding that dopexamine action is based on receptors activation, one can likely expect a quicker arterial flow reduction than that for acetate, which has to be metabolized by the intestine tissue to locally produces adenosine.

Increasing dose cohorts starting from 3 up to 10 μg/kg/min infusion should be investigated. Escalation trial can be ended before the maximal rate, when no more additional T/N improvement is observed.

Regarding ethical considerations, one has to note that the patients participating in the trials could likely have their therapy improved versus current radioembolization state-of-the-art.

## 7. Discussion

Clinical studies [10] showed that arterial flow reduction using intra-arterial vasoconstrictor infusion was limited in intensity and in duration by the HABR and by the hepatic vascular escape mechanism, resulting in a twofold hepatic arterial flow reduction for only a few minutes (Figure 1). On the contrary, HABR triggered exhibited higher hepatic arterial flow reduction during a longer period, which is more compatible with a therapeutic session, especially regarding when tip re-positioning is required. To the best of our knowledge, HABR triggering has never been investigated in the purpose of increasing the T/N.

Table 3 shows the pros and cons of different agents. The expected T/N improvement takes into account that, due to the blood redistribution resulting from the hepatic tissue arterial flow reduction, the tumor blood flow could be increased by a factor of about 1.5 (Figure 1a).

Liquid meal has the benefit to have no adverse side effect, but the maximal arterial flow timing being about 30 min and the absence of duration control discard its use. Furthermore, a gastric going back to the respiratory track could be unsafe. Sodium acetate is contraindicated in patients with impaired liver function often present in advanced HCC stages. 

Dopexamine has the benefit to have no hepatic-based contraindications, to have a fourfold prompt arterial flow reduction which will be sustained during the whole infusion duration. Dopexamine experience in cardiac failure management proved it is safe at a dose rate twofold higher than that producing the observed fourfold reduction and during infusion time two orders of magnitude higher than that needed in liver radioembolization.

Standard liver radioembolization in HCC provided a T/N of about 2.1 ± 1.3 [2], meaning that numerous patients have a T/N just a little bit higher than unity, strongly limiting the dose deliverable to the tumors. More dramatic, a recent study [64] showed that about 60% of patients have a least one tumor exhibiting a T/N lower than unity, making it impossible to cure these patients. The expected T/N improvement using dopexamine should clear these limitations.

The actual dopexamine T/N improvement assessment can be performed during the preliminary arteriography with MAA injection, always done a few weeks before each therapeutic radioembolization in order to exclude extra-hepatic uptake, such as lung shunt, and to plan the ^90^Y activity to be injected. Escalation cohorts can be performed starting with the dose rate showing the fourfold reduction in animal studies, i.e., 4 μg/kg/min infusion, up to the safe limit observed for very long infusions in humans, i.e., 10 μg/kg/min infusion. Obviously, dose rate escalation should be stopped when no more additional T/N improvement is observed versus the previous cohort.

Regarding ethics issues, the additional act is limited to the injection of a safe and well-known drug. The additional dose provided by the other radionuclide injections is about four orders of magnitude lower than the dose received during the therapy session. A positive feature is that the patients enrolled in the trial will likely benefit from an improved therapy versus the current state-of-the-art one. Another important feature is that the trial objective complies with the international committee on radiobiological protection (ICRP) recommendations [65] and with the European council directive [66], i.e., to implement any measure allowing the reduction of the dose to the normal tissues while preserving the therapy intent to the target tissues. 

## 8. Conclusions

The liver has the unique feature of being made of about one million hexagonal lobules fed at each corner by an arteriole and a portal venule. Within each lobule, the sophisticated HABR mechanism acts on the arteriole diameter in order to maintain constant the total lobule blood flow. Dopexamine infusion has been evidenced to increase by a factor of two the portal flow; in return, the HABR dramatically reduces the arterial flow during the whole dopexamine infusion. A four to sixfold improvement of the tumor to normal tissue ratio (T/N) is expected, pushing the radioembolization to a real cure intent. 

## Figures and Tables

**Table 1:** Dose rates and durations investigated in healthy volunteers.

Ref.	*n*	Age (Year)	Status	Rate (μmol·kg^−1^·min^−1^)	Duration (min)	Total (μmol·kg^−1^)
[23]	6	21–30	healthy	20	180	3600
[24]	9	30 ± 1	healthy	36	120	4320
[25]	7	54 ± 4	coronary disease	60	20	1200
[26]	8	23 ± 1	healthy	52	60	3120
[27]	8	25 ± 1	healthy	125	40	5000

**Table 2:** Organ blood flow in mL/min/g in dogs during different dopexamine infusion rates extracted from [36].

Organ	Control	1.1 μg/kg/min	3.6 μg/kg/min	11 μg/kg/min
Stomach	0.47 ± 0.14	1.03 ± 0.23	1.21 ± 0.15	1.82 ± 0.32
Spleen	1.19 ± 0.15	2.02 ± 0.31	1.26 ± 0.12	1.06 ± 0.21
Small int.	1.55 ± 0.19	2.40 ± 0.38	1.80 ± 0.17	2.16 ± 0.18
Large int.	0.66 ± 0.08	0.77 ± 0.12	0.86 ± 0.16	1.31 ± 0.31
Liver (artery)	0.08 ± 0.03	0.08 ± 0.02	0.02 ± 0.00	0.04 ± 0.01
Kidney	4.23 ± 0.46	5.68 ± 0.48	5.82 ± 0.35	5.89 ± 0.99

**Table 3:** Pros and cons of different agents.

Agent	T/N	Pro	Con
AT-II	×3	prompt	limited duration
liquid meal	×4	no adverse effect	delay, duration
sodium acetate	×4	prompt, duration	sodium load in cirrhosis
dopexamine	×6	prompt, duration	-

## References

[1] Padia S.A., Lewandowski R.J., Johnson G.E., Sze D.Y., Ward T.J., Gaba R.C., Baerlocher M.O., Gates V.L., Riaz A., Brown D.B. (2017). Society of Interventional Radiology Standards of Practice Committee: Radioembolization of hepatic malignancies: Background, quality improvement guidelines, and future directions. J. Vasc. Interv. Radiol..

[2] Ilhan H., Goritschan A., Paprottka P., Jakobs T.F., Fendler W.P., Todica A., Bartenstein P., Hacker M., Haug A.R. (2015). Predictive value of 99mTc-MAA SPECT for 90Y-labeled resin microsphere distribution in radioembolization of primary and secondary hepatic tumors. J. Nucl. Med..

[3] Walrand S., Hesse M., Chiesa C., Lhommel R., Jamar F. (2014). The low hepatic toxicity per Gray of 90Y glass microspheres is linked to their transport in the arterial tree favoring a nonuniform trapping as observed in posttherapy PET imaging. J. Nucl. Med..

[4] Garin E., Tselikas L., Guiu B., Chalaye J., Edeline J., de Baere T., Assenat E., Tacher V., Robert C., Terroir-Cassou-Mounat M. (2020). Personalised versus standard dosimetry approach of selective internal radiation therapy in patients with locally advanced hepatocellular carcinoma (DOSISPHERE-01): A randomised, multicentre, open-label phase 2 trial. Lancet Gastroenterol. Hepatol..

[5] Sangro B., Iñarrairaegui M., Bilbao J.I. (2012). Radioembolization for hepatocellular carcinoma. J. Hepatol..

[6] Goldberg J.A., Murray T., Kerr D.J., Willmott N., Bessent R.G., McKillop J.H., McArdle C.S. (1991). The use of angiotensin II as a potential method of targeting cytotoxic microspheres in patients with intrahepatic tumour. British journal of cancer. Br. J. Cancer.

[7] Goldberg J.A., Thomson J.A., Bradnam M.S., Fenner J., Bessent R.G., McKillop J.H., Kerr D.J., McArdle C.S. (1991). Angiotensin II as a potential method of targeting cytotoxic-loaded microspheres in patients with colorectal liver metastases. Br. J. Cancer.

[8] Sasaki Y., Imaoka S., Hasegawa Y., Nakano S., Ishikawa O., Ohigashi H., Taniguchi K., Koyama H., Iwanaga T., Terasawa T. (1985). Changes in distribution of hepatic blood flow induced by intra-arterial infusion of angiotensin II in human hepatic cancer. Cancer.

[9] Macedo M.P., Lautt W.W. (1998). Shear-induced modulation of vasoconstriction in the hepatic artery and portal vein by nitric oxide. Am. J. Physiol. Gastrointest. Liver Physiol..

[10] Lautt W.W. (2009). Hepatic Circulation: Physiology and Pathophysiology. Colloquium Series on Integrated Systems Physiology: From Molecule to Function.

[11] Van den Hoven A.F., Smits M.L., Rosenbaum C.E., Verkooijen H.M., van den Bosch M.A., Lam M.G. (2014). The effect of intra-arterial angiotensin II on the hepatic tumor to non-tumor blood flow ratio for radioembolization: A systematic review. PLoS ONE.

[12] Gray B., Van Hazel G., Hope M., Burton M., Moroz P., Anderson J., Gebski V. (2001). Randomised trial of SIR-Spheres® plus chemotherapy vs. chemotherapy alone for treating patients with liver metastases from primary large bowel cancer. Ann. Oncol..

[13] Van Hazel G., Blackwell A., Anderson J., Price D., Moroz P., Bower G., Cardaci G., Gray B. (2004). Randomised phase 2 trial of SIR-Spheres® plus fluorouracil/leucovorin chemotherapy versus fluorouracil/leucovorin chemotherapy alone in advanced colorectal cancer. J. Surg. Oncol..

[14] Dhabuwala A., Lamerton P., Stubbs R.S. (2005). Relationship of 99m technetium labelled macroaggregated albumin (99m Tc-MAA) uptake by colorectal liver metastases to response following Selective Internal Radiation Therapy (SIRT). BMC Nucl. Med..

[15] Stubbs R.S., Cannan R.J., Mitchell A.W. (2001). Selective internal radiation therapy with 90yttrium microspheres for extensive colorectal liver metastases. J. Gastrointest. Surg..

[16] Stubbs R.S., O’Brien I., Correia M.M. (2006). Selective internal radiation therapy with 90Y microspheres for colorectal liver metastases: Single-centre experience with 100 patients. ANZ J. Surg..

[17] Boppudi S., Wickremesekera S.K., Nowitz M., Stubbs R. (2006). Evaluation of the role of CT in the assessment of response to selective internal radiation therapy in patients with colorectal liver metastases. Australas. Radiol..

[18] Dauzat M., Lafortune M., Patriquin H., Pomier-Layrargues G. (1994). Meal induced changes in hepatic and splanchnic circulation: A noninvasive Doppler study in normal humans. Eur. J. Appl. Physiol. Occup. Physiol..

[19] Carmichael F.J., Saldivia V., Varghese G.A., Israel Y., Orrego H. (1988). Ethanol-induced increase in portal blood flow: Role of acetate and A1-and A2-adenosine receptors. Am. J. Physiol. Gastrointest. Liver Physiol..

[20] Buyer D.R., Krahmer R.L., Lau A.H., Wang H.C., Ferguson J.L., Daugirdas J.T. (1993). Regional blood flow redistribution due to acetate. J. Am. Soc. Nephrol..

[21] Liang C.S., Lowenstein J.M. (1978). Metabolic control of the circulation: Effects of acetate and pyruvate. J. Clin. Investig..

[22] Kirkendol P.L., Robie N.W., Gonzalez F.M., Devia C.J. (1978). Cardiac and vascular effects of infused sodium acetate in dogs. ASAIO J..

[23] Chioléro R., Mavrocordatos P., Burnier P., Cayeux M.C., Schindler C., Jéquier E., Tappy L. (1993). Effects of infused sodium acetate, sodium lactate, and sodium beta-hydroxybutyrate on energy expenditure and substrate oxidation rates in lean humans. Am. J. Clin. Nutr..

[24] Burnier P., Tappy L., Jéquier E., Schneeberger D., Chioléro R. (1992). Metabolic and respiratory effects of infused sodium acetate in healthy human subjects. Am. J. Physiol..

[25] Nitenberg A., Huyghebaert M.F., Blanchet F., Amiel C. (1984). Analysis of increased myocardial contractility during sodium acetate infusion in humans. Kidney Int..

[26] Evans M.K., Savasi I., Heigenhauser G.J., Spriet L.L. (2001). Effects of acetate infusion and hyperoxia on muscle substrate phosphorylation after onset of moderate exercise. Am. J. Physiol. Endocrinol. Metab..

[27] Putman C.T., Spriet L.L., Hultman E., Dyck D.J., Heigenhauser G.J. (1995). Skeletal muscle pyruvate dehydrogenase activity during acetate infusion in humans. Am. J. Physiol..

[28] Sodium Acetate Injection USP. https://www.accessdata.fda.gov/drugsatfda_docs/label/2014/018893s025lbl.pdf.

[29] Mansell M.A., Morgan S.H., Moore R., Kong C.H., Laker M.F., Wing A.J. (1987). Cardiovascular and Acid—Base Effects of Acetate and Bicarbonate Haemodialysis. Nephrol. Dial. Transplant..

[30] Richards R.H., Vreman H.J., Zager P., Feldman C., Blaschke T., Weiner M.W. (1982). Acetate metabolism in normal human subjects. Am. J. Kidney Dis..

[31] Burggraaf J., Schoemaker R.C., Kroon J.M., Cohen A.F. (1998). The influence of nifedipine and captopril on liver blood flow in healthy subjects. Br. J. Clin. Pharm..

[32] Ljung B. (1990). Vascular selectivity of felodipine: Experimental pharmacology. J. Cardiovasc. Pharm..

[33] Bengtsson-Hasselgren B., Rönn O., Blychert L.O., Edgar B., Raner S. (1990). Acute effects of felodipine and nifedipine on hepatic and forearm blood flow in healthy men. Eur. J. Clin. Pharm..

[34] Ota K., Shijo H., Kokawa H., Kubara K., Kim T., Akiyoshi N., Yokoyama M., Okumura M. (1995). Effects of nifedipine on hepatic venous pressure gradient and portal vein blood flow in patients with cirrhosis. J. Gastroenterol. Hepatol..

[35] Bartsch S., Brüning A., Reimann F.M., Ludwig D. (2004). Haemodynamic effects of dopexamine on postprandial splanchnic hyperaemia. Eur. J. Clin. Investig..

[36] Biro G.P., Douglas J.R., Keon W.J., Taichman G.C. (1988). Changes in regional blood flow distribution induced by infusions of dopexamine hydrochloride or dobutamine in anesthetized dogs. Am. J. Cardiol..

[37] Tobata D., Takao K., Mochizuki M., Nishimura R., Sasaki N. (2004). Effects of dopamine, dobutamine, amrinone and milrinone on regional blood flow in isoflurane anesthetized dogs. J. Vet. Med. Sci..

[38] Fitton A., Benfield P. (1990). Dopexamine hydrochloride. Drugs.

[39] Tanaka M., Iwakiri Y. (2016). The hepatic lymphatic vascular system: Structure, function, markers, and lymphangiogenesis. Cell. Mol. Gastro. Hepa..

[40] Richter S., Mücke I., Menger M.D., Vollmar B. (2000). Impact of intrinsic blood flow regulation in cirrhosis: Maintenance of hepatic arterial buffer response. Am. J. Physiol. Gastrointest. Liver Physiol..

[41] Gülberg V., Haag K., Rössle M., Gerbes A.L. (2002). Hepatic arterial buffer response in patients with advanced cirrhosis. Hepatology.

[42] Roldán-Alzate A., Frydrychowicz A., Said A., Johnson K.M., Francois C.J., Wieben O., Reeder S.B. (2015). Impaired regulation of portal venous flow in response to a meal challenge as quantified by 4D flow MRI. J. MRI.

[43] Desmorat H., Vinel J.P., Lahlou O., Pipy B., Badia P., Cales P., Combis J.M., Souqual M.C., Pascal J.P. (1991). Systemic and splanchnic hemodynamic effects of molsidomine in rats with carbon tetrachloride–induced cirrhosis. Hepatology.

[44] Peschl L. (1978). Clinical and experimental investigations of the effect of dopamine on haemodynamics and function of kidney and liver. Wiener Klinische Wochenschrift. Supplementum.

[45] Sansoè G., Ferrari A., D’Alimonte P., Trenti T., Zoboli P., Romagnoli R., Villa E., Manenti F. (1998). Beneficial hemodynamic effects of dipyridamole on portal circulation in cirrhosis. Am. J. Gastrol..

[46] Dinç H., Kaplcloglu S., Cihanyurdu N., Çan G., Ünal M., Topkaya L., Gümele H.R. (1996). Effect of verapamil on portal and splanchnic hemodynamics in patients with advanced posthepatitic cirrhosis using duplex Doppler ultrasound. Eur. J. Radiol..

[47] Hadengue A., Moreau R., Bacq Y., Gaudin C., Braillon A., Lebrec D. (1991). Selective dopamine DA1 stimulation with fenoldopam in cirrhotic patients with ascites: A systemic, splanchnic and renal hemodynamic study. Hepatology.

[48] Shiraha H., Iwamuro M., Okada H. (2020). Hepatic stellate cells in liver tumor. Adv. Exp. Med. Biol..

[49] Yang Z.F., Poon R.T. (2008). Vascular changes in hepatocellular carcinoma. Anat. Rec..

[50] Thabut D., Shah V. (2010). Intrahepatic angiogenesis and sinusoidal remodeling in chronic liver disease: New targets for the treatment of portal hypertension?. J. Hepatol..

[51] Yamamoto T., Hirohashi K., Kaneda K., Ikebe T., Mikami S., Uenishi T., Kanazawa A., Takemura S., Shuto T., Tanaka H. (2001). Relationship of the microvascular type to the tumor size, arterialization and dedifferentiation of human hepatocellular carcinoma. Jpn. J. Cancer Res..

[52] Mortelé K.J., Mortele B., Silverman S.G. (2004). CT features of the accessory spleen. Am. J. Roentgenol..

[53] Bajwa S.A., Kasi A. (2020). Anatomy, Abdomen and Pelvis, Accessory Spleen. StatPearls.

[54] Gill N., Nasir A., Douglin J., Pretterklieber B., Steinke H., Pretterklieber M., Cotofana S. (2017). Accessory spleen in the greater omentum: Embryology and revisited prevalence rates. Cells Tissues Organs..

[55] Xuan Z., Chen J., Song P., Du Y., Wang L., Wan D., Zheng S. (2018). Management of intrahepatic splenosis: A case report and review of the literature. World J. Surg. Oncol..

[56] Serra M., Columbano A., Perra A., Kowalik M.A. (2020). Animal Models: A Useful Tool to Unveil Metabolic Changes in Hepatocellular Carcinoma. Cancers.

[57] Subramanian S., Pandey U., Chaudhari P., Tyagi M., Gupta S., Singh G., Dash A., Samuel G., Venkatesh M. (2016). Preliminary evaluation of indigenous 90Y-labelled microspheres for therapy of hepatocellular carcinoma. Ind. J. Med. Res..

[58] Wilkins L.R., Brautigan D.L., Wu H., Yarmohammadi H., Kubicka E., Serbulea V., Leitinger N., Liu W., Haaga J.R. (2017). Cinnamic acid derivatives enhance the efficacy of transarterial embolization in a rat model of hepatocellular carcinoma. Cardiovasc. Interv. Radiol..

[59] Becker S., Ardisson V., Lepareur N., Sergent O., Bayat S., Noiret N., Gaboriau F., Clément B., Boucher E., Raoul J.L. (2010). Increased Lipiodol uptake in hepatocellular carcinoma possibly due to increased membrane fluidity by dexamethasone and tamoxifen. Nucl. MedBbiol..

[60] Zhou B., Wang J., Yan Z. (2014). Ginsenoside Rg3 attenuates hepatoma VEGF overexpression after hepatic artery embolization in an orthotopic transplantation hepatocellular carcinoma rat model. Oncotargets Ther..

[61] Ni J.Y., Xu L.F., Wang W.D., Huang Q.S., Sun H.L., Chen Y.T. (2017). Transarterial embolization combined with RNA interference targeting hypoxia-inducible factor-1α for hepatocellular carcinoma: A preliminary study of rat model. J. Cancer Res. Clin. Oncol..

[62] Kao Y.H., Tan E.H., Teo T.K., Ng C.E., Goh S.W. (2011). Imaging discordance between hepatic angiography versus Tc-99m-MAA SPECT/CT: A case series, technical discussion and clinical implications. Ann. Nucl. Med..

[63] Bowen S.R., Chapman T.R., Borgman J., Miyaoka R.S., Kinahan P.E., Liou I.W., Sandison G.A., Vesselle H.J., Nyflot M.J., Apisarnthanarax S. (2016). Measuring total liver function on sulfur colloid SPECT/CT for improved risk stratification and outcome prediction of hepatocellular carcinoma patients. EJNMMI Res..

[64] Smits M.L., Elschot M., van den Bosch M.A., van de Maat G.H., van het Schip A.D., Zonnenberg B.A., Seevinck P.R., Verkooijen H.M., Bakker C.J., de Jong H.W. (2013). In vivo dosimetry based on SPECT and MR imaging of 166Ho-microspheres for treatment of liver malignancies. J. Nucl. Med..

[65] Yonekura Y., Mattsson S., Flux G., Bolch W.E., Dauer L.T., Fisher D.R., Lassmann M., Palm S., Hosono M., Doruff M. (2019). ICRP publication 140: Radiological protection in therapy with radiopharmaceuticals. Ann. ICRP.

[66] Directive 2013/59/Euratom. https://eur-lex.europa.eu/LexUriServ/LexUriServ.do?uri=OJ:L:2014:013:0001:0073:EN:PDF.

